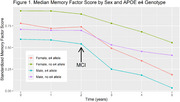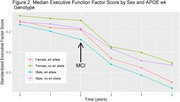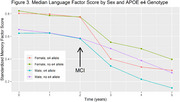# Cognitive Trajectories in Parkinson's Disease and Lewy Body Disease

**DOI:** 10.1002/alz70860_106929

**Published:** 2025-12-23

**Authors:** Julia W. Gallini, Santiago Gutierrez Gomez, Ashita S. Gurnani, Seo‐Eun Choi, Shubhabrata Mukherjee, Emily H. Trittschuh, Timothy J. Hohman, Andrew J. Saykin, Paul K Crane, Yorghos Tripodis, Jesse Mez, Ludy Shih

**Affiliations:** ^1^ Boston University School of Public Health, Boston, MA, USA; ^2^ Boston University Chobanian and Avedisian School of Medicine, Boston, MA, USA; ^3^ Boston University Chobanian & Avedisian School of Medicine, Boston, MA, USA; ^4^ Department of Medicine, University of Washington, Seattle, WA, USA; ^5^ University of Washington, Seattle, WA, USA; ^6^ University of Washington, School of Medicine, Seattle, WA, USA; ^7^ Vanderbilt Memory and Alzheimer's Center, Vanderbilt University School of Medicine, Nashville, TN, USA; ^8^ Indiana University School of Medicine, Indianapolis, IN, USA; ^9^ University of Washington School of Medicine, Seattle, WA, USA; ^10^ Beth Israel Deaconess Medical Center, Harvard Medical School, Boston, MA, USA

## Abstract

**Background:**

Parkinson's disease (PD) is the second most common neurodegenerative illness after Alzheimer's disease. Dementia with Lewy bodies (DLB) shares clinical and neuropathological features with PD and requires cognitive impairment to meet clinical criteria. Although cognitive impairment is common in both PD and DLB, rates of cognitive decline can vary markedly, and associated predictors are poorly understood. This study aimed to characterize the effects of risk factors‐ particularly APOE and sex‐ on the trajectories of cognitive factor scores for memory, executive function, and language in persons with PD and DLB.

**Methods:**

Using National Alzheimer's Coordinating Center data, we identified participants with PD or DLB with a cognitively normal neuropsychological baseline and at least two total cognitive assessments. Factor scores were previously derived for language, memory and executive function. We fit three linear mixed effects models, one each for memory, executive function, and language factor scores. Predictors in these models included sex, race, baseline age, *APOE* genotype (1‐2 vs. 0 ε4 alleles), education, time from baseline, and interactions between sex and time and *APOE* and time. We also included a linear spline at the first diagnosis of mild cognitive impairment (MCI) or dementia (some participants remained cognitively normal throughout).

**Results:**

494 participants with PD or DLB [193 (39.1%) female, mean baseline age: 71 (8.7 SD), 118 (23.9%) with 1‐2 ε4 alleles] were included. In the memory model, there was a faster rate of cognitive decline post‐MCI diagnosis (0.04 SD/yr; *p* = 0.001). This post‐MCI effect was significantly modified by *APOE* genotype (*p* = 0.01) but not by sex (*p* = 0.07). Post‐MCI diagnosis, people with 1‐2 *APOE* ε4 alleles declined 0.05 SD/year faster than people with 0 ε4 alleles. In the executive function and language models there was a faster rate of cognitive decline post‐MCI diagnosis (0.03 SD/yr, *p* = 0.02; 0.05 SD/yr, *p* <0.001) but these effects were not modified by *APOE*.

**Conclusions:**

*APOE* genotype was associated with an increased rate of memory decline, but not executive function or language decline in participants with PD/DLB after an MCI diagnosis. This factor may be useful for prognostication and aid in a personalized medicine approach to PD/DLB.